# IgA anti-Actin antibodies in children with celiac disease: comparison of immunofluorescence with Elisa assay in predicting severe intestinal damage

**DOI:** 10.1186/1824-7288-36-25

**Published:** 2010-03-18

**Authors:** Elena Bazzigaluppi, Barbara Parma, Giulia M Tronconi, Patrizia Corsin, Luca Albarello, Stefano Mora, Graziano Barera

**Affiliations:** 1 Diagnostica e Ricerca San Raffaele S.p.A. LaboRaf, San Raffaele Scientific Institute, Via Olgettina 60, Milan, 20132, Italy; 2 Department of Pediatrics, San Raffaele Scientific Institute, Via Olgettina 60, Milan, 20132, Italy; 3 Division of Surgical Pathology, San Raffaele Scientific Institute, Via Olgettina 60, Milan, 20132, Italy; 4 Laboratory of Pediatric Endocrinology and BoNetwork, San Raffaele Scientific Institute, Via Olgettina 60, Milan, 20132, Italy

## Abstract

**Background:**

Previous studies have demonstrated that the presence of serum IgA antibodies against actin filaments (AAA) in patients with celiac disease (CD) is strongly associated with mucosal damage and severe degrees of villous atrophy.

The aims of the present study were (1) to verify the effectiveness of IgA-AAA in newly diagnosed CD patients in a clinical setting (2) to compare the immunofluorescence assay with ELISA assay; (3) to compare the correlation of our IgA anti-tissue transglutaminase antibodies (tTG-Ab) class with mucosal intestinal lesions.

**Methods:**

90 patients underwent endoscopy and multiple biopsies for suspected CD on the basis of symptoms, in presence of positive tTG-Ab tests. Twenty biopsied and 25 not-biopsied subjects with negative tTG-Ab were tested as control groups.

IgA-AAA assays were performed by indirect immunofluorescence using rat epithelial intestinal cells, and by ELISA with a commercial kit. tTG-Ab assay was a radio-binding assay.

Intestinal specimens were collected by upper endoscopy and the histological study was done according to the Marsh's classification modified by Oberhuber (M/O). Auto-antibodies assays and histological evaluation have been performed blindly by skilled operators.

**Results:**

CD diagnosis was confirmed in 82 patients (type I M/O in 2 patients, IIIA in 18 patients, IIIB in 29 patients and IIIC in 33 patients). Two patients with type 1 lesion in presence of positive tTG-Ab and abdominal complaints, started a gluten free diet.

The rate of IgA-AAA positivity (sensitivity) by IFI and ELISA in histologically proven celiac disease patients, were 5.5% and 25% patients in IIIA, 27.5% and 34.4% patients in IIIB, 78.8% and 75% in IIIC patients, respectively.

Patients with normal or nearly normal mucosa, regardless of tTG-Ab status, presented negative IgA-AAA IFI assay. On the other hand, 1 patient with normal mucosa but positive tTG-Ab, also presented positive IgA-AAA ELISA. All healthy non biopsied controls had negative IgA-AAA. tTG-Ab serum concentration was significantly correlated with more severe intestinal lesion (IIIB, IIIC M/O).

**Conclusions:**

IgA-AAA may be undetectable in presence of severe mucosal damage. Histology is still necessary to diagnose celiac disease and IgA-AAA cannot be included in usual screening tests, because it has little to offer if compared to the well-established tTG-Ab.

IgA-AAA could be an adjunctive, very useful tool to support the diagnosis of CD in case of suboptimal histology, when the biopsy is to be avoided for clinical reasons, or in case of negative parents' consensus.

## Background

Celiac disease (CD) is a permanent, immune-mediated enteropathy caused by gluten ingestion in genetically susceptible subjects. It is characterized by various degrees of villous atrophy in presence of gluten-dependent autoantibodies [1,2].

The prevalence of CD is currently increasing compared to our experience in the past. Serological findings, such as anti-endomysium (EmA) and anti-tissue-transglutaminase antibodies (tTG-Ab), are very useful in increasing our diagnostic capacity [3-5], but are not always able to predict the histological features [6-8].

The pathogenic cascade that causes the typical histological lesions, characterized by flat mucosa with tissue destruction and reorganization of the intestinal picture, is still partially unknown. In this respect, a role of cytoskeleton has been described: the gluten ingestion has been reported to induce a rapid alteration of the actin network on intestinal mucosa of CD patients [9]. Gliadin rapidly increases actin polymerization leading to rearrangement of actin filaments, especially in the intracellular subcortical compartment [10]. It is likely that newly generated actin polymers may be exposed to gut-associated lymphatic tissue, causing the production of IgA antibodies against actin filaments (IgA-AAA).

Previous studies have described that the presence of antibodies against actin filaments is associated with severe degrees of mucosal damage and that IgA-AAA may also contribute to exacerbate the villous' cytoskeleton damage [11-14]. It has also been suggested that the presence of IgA-AAA may, in some patients, overcome the need of the intestinal biopsy [9].

The aims of this study were to evaluate, using two different assays (immunofluorescence (IFI) and ELISA), the prevalence of IgA-AAA in a group of newly diagnosed CD patients and to verify the relationship between these serological tests and degrees of intestinal lesions. Finally, we verified the reliability of our tTG-Ab IgA test in predicting intestinal mucosal damage.

## Methods

### Patients

We enrolled between November 2006 and March 2008:

- 90 patients (59 F, 31 M, age mean ± SD: 6.8 ± 4.1 yrs), who performed endoscopy and multiple biopsies for suspected CD, on the basis of symptoms and positive tTG-Ab. Twenty patients had a typical presentation, characterized by gastrointestinal complaints (malabsorption syndrome, abdominal pain, prominent abdomen), 34 patients had non-intestinal presentation (anemia, failure to thrive, dermatitis), and 36 patients were identified during screening program in at risk groups (type I diabetes, autoimmune disease, first degree relatives of CD);

- 45 control subjects, matched for age and sex, with negative tTG-Ab tests: 20 underwent endoscopy for persistent GI symptoms (dyspepsia in 12; recurrent abdominal pain in 8 patients).

Informed consent to the study was obtained from all patients and control subjects' parents or legal guardians. The study was performed in accordance to the principles of the Declaration of Helsinki.

### Autoantibodies measurements

**Figure 1:**
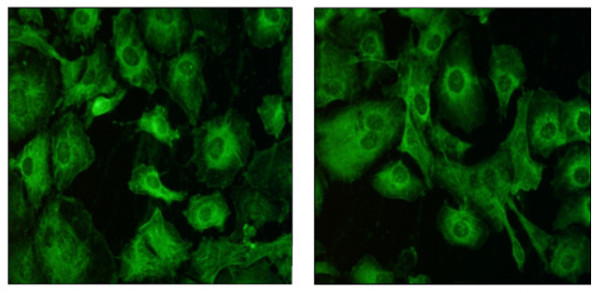
**Fluorescence images at microscopy (400×)**. The positive assay is represented by fluorescent cytoplasmatic parallel actin filaments on rat epithelial intestinal cells.

- IgA and IgG antibodies to recombinant human tissue transglutaminase C were measured by radio-binding assay as previously described [15]. Results for each assay were expressed as arbitrary units derived from standard curves of serial dilutions of a serum with both IgA and IgG tTG tested in each assay [ranges tTG IgA 0-1.3 AU; tTG IgG 0-8 AU].

- IgA-AAA were evaluated by indirect immunofluorescence on sections containing rat epithelial intestinal cells (Eurospital, Trieste-Italy). A 1:5 dilution of serum sample from each patient and positive control were incubated at 56°C for 30 min. The slides were examined under a fluorescence microscope (Leitz Laborlux) using 250-400× magnifications (Figure 1). Fluorescence was compared with positive and negative control samples tested in each assay.

- The detection of IgA AAA by ELISA was conduced using a commercially available assay (Quanta Lite™ F-actin IgA INOVA, 704500) [13]. The threshold was: positive more than 25 U, border-line 20.1-24.9, negative ≤ 20.

Autoantibodies assays have been performed blindly by a single skilled operator (E.B.).

### Intestinal histology

We acquired biopsy specimens in the 4 quadrant of the second part of the duodenum, on the first fold distal to the papilla of Vater, according to the guidelines for the diagnosis of CD [16]. The histological specimens were fixed in 10% formalin and stained with hematoxylin and eosin. The biopsy's grading was made according to the Marsh's classification modified by Oberhuber et al (M/O) [17].

Histological evaluations have been performed blindly by a single skilled operator (L.A.).

### Diagnosis of CD

Diagnosis of CD was made according to the following criteria:

- histological lesions type II or III according to M/O, in presence of tTG-Ab regardless of intestinal symptoms;

- minimal mucosal lesions (type I M/O) in presence of tTG-Ab positivity and gluten sensitivity with clinical manifestations.

### Statistical analysis

The distribution of categorical variables has been evaluated by chi-square test.

The differences between patients with negative or positive IgA-AAA IFI/ELISA have been determined by unpaired t-test.

The differences of tTG-Ab values between patients grouped according to biopsies grading have been evaluated by one-way analysis of variance (ANOVA).

Data are shown as mean ± SD, unless otherwise stated.

## Results

CD diagnosis was confirmed in 82 subjects: 18 patients presented IIIA lesion, 29 patients presented IIIB lesion and 33 patients had IIIC lesion; two patients had type 1 lesion in presence of positive tTG-Ab and abdominal complaints. The gastrointestinal manifestations of the latter patient can be referred to as minimal immunopathological changes in the intestine exposed to gluten even in the absence of overt CD enteropathy: this condition is called "gluten sensitivity" and is defined by some morphological, immunological, or functional disorder that generally respond to gluten exclusion [18]. All patients started a gluten free diet following the diagnosis.

Eight patients with positive tTG-Ab presented normal mucosa (type 0 M/O). All 20 biopsied controls with negative tTG-Ab had normal histology. The tTG-Ab serum concentration of patients with IIIC M/O (66.14 ± 33.8 AU) or IIIB M/O (53.16 ± 30.76 AU) were higher than IIIA intestinal lesion (32.36 ± 29.98 AU, F = 3.4; p = 0.0123). The 8 patients with normal mucosa and the two ones with minor lesions (type I M/O) presented a low tTG-Ab titre (15.85 ± 11.50 AU).

IgA-AAA tested by IFI and ELISA were detected in 35 (43.7%) and in 38 (47.5%) untreated celiac patients, respectively.

The rate of IgA-AAA positivity by IFI and ELISA in histologically proven celiac disease patients, were 5.5% and 25% patients in IIIA, 27.5% and 34.4% patients in IIIB, 78.8% and 75% in IIIC patients, respectively: showing an high sensitivity in predicting severe intestinal damage.

IgA-AAA were not detectable in control subjects; just one biopsied subject resulted positive to IgA-AAA tested by ELISA.

Positivity rate to IgA-AAA was different according to histological degree of lesion, as explained in table 1.

**Table 1:** IgA-AAA positivity by IFI and ELISA in biopsied controls and CD subjects.

Marsh Oberhuber classification						
**Mucosa Histopathology**	**Type 0** **(n = 28)**		**Type I** **(n = 2)**	**Type IIIA** **(n = 18)**	**Type III B** **(n = 29)**	**Type III C** **(n = 33)**
					
	**Positive tTG-Ab** **(n = 8)**	**Negative tTG-Ab** **(n = 20)**				

**Positive** **IgA-AAA IFI (%)**	0	0	0	1 (5.5%)	8 (27.5%)	26 (78.8%)

**Positive** **IgA-AAA ELISA (%)**	1 (3.3%)	0	0	4 (25%)	11 (39.3%)	24 (75%)

Sensitivity, sensibility, negative and positive predictive values of IgA-AAA tested by IFI and ELISA are shown in table 2. We found that subjects with negative IgA-AAA IFI test had significantly lower IgA-AAA concentration measured by ELISA, compared to subjects with positive results by IFI (Wilcoxon Z: 4.89, p < 0.0001).

**Table 2:** Sensitivity, specificity, negative predictive value, positive predictive value of IFI and ELISA methods of IgA-AAA

	Sensitivity	Specificity	Negative predictive value	Positive predictive value
**IgA-AAA IFI**	42,68%	100%	37%	100%

**IgA-AAA ELISA**	47,56%	96,43%	38,57%	97,50%

**Figure 2:**
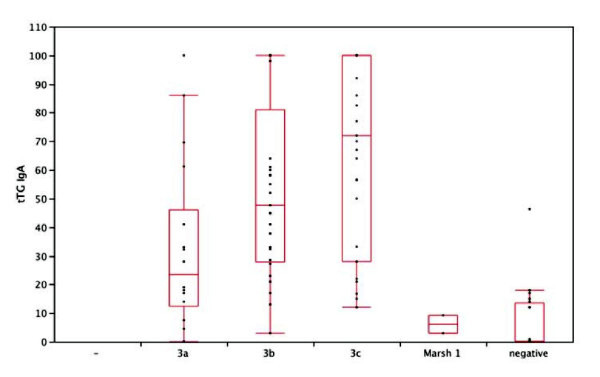
**tTG-Ab titres in different mucosal lesions' degrees (p = 0.0021)**.

tTG-Ab assay was plotted against mucosal lesion degree and results are shown in figure 2 (Significant differences between groups were shown by analysis of variance F = 6.7; p = 0.0021).

## Discussion

The diagnosis of celiac disease relies on the demonstration of intestinal histological lesions associated with presence of positive gluten-autoantibodies and or clinical improvement after gluten free diet.

Endoscopic biopsy is an useful tool, but it is often perceived as invasive and dangerous. IgA-AAA has been proposed as a serological marker of intestinal mucosa damage as it seems to correlate with histological lesions.

Recent studies suggest that IgA-AAA test could be considered a useful tool in diagnosis of CD, being a reliable marker of severe intestinal mucosal damage. In this respect the immunofluorescence assay has been suggested as an useful method and ELISA has been demonstrated to be an accurate assay for their determination [11-13].

To add our experience to these data, we studied IgA-AAA antibodies by two different assays (IFI and ELISA), that resulted positive in a similar proportion in newly diagnosed CD patients (43.7% and 47.5%, respectively).

Our data confirm that serum IgA-AAA were more frequently positive in presence of total villous atrophy than in patients with subtotal or mild villous atrophy. Patients with normal mucosa, regardless of tTG-Ab status, presented negative IgA-AAA by IFI assay, thus showing a specificity of 100%.

Because previous reports [19-22] indicated a possible association between IgA-AAA positivity and inflammatory bowel disease or autoimmune/infective hepatic disorders, all patients were investigated for inflammatory indices and hepatic function. None of the tested subject had altered biochemical indices.

These results are apparently in contrast with data previously described in literature [11] that showed higher sensitivity of IgA-AAA due to the presence of IgA-AAA reactivity also in subtotal mild mucosal involvement. This discordance could be explained by some technical bias related to measurements methods, as recently reported [23]. It has been underlined that the sensitivity of IgA-AAA assay can significantly be enhanced by heating or chelating with calcium the serum samples before performing IgA-AAA test. We did not perform such treatment because we strictly followed the manufacturer's instructions.

Although IFI assays are operator-dependent techniques, we believe that this case does not apply to our study because the operator that performed IFI test is a skilled one and the results obtained by IFI are not different from data obtained by ELISA.

On the other hand, tTG-Ab were able to distinguish patients with severe lesions (M/O type 3) from those with milder ones (M/O type 0-1); as recently described [23,24], we can confirm that IgA-AAA assay does not add to tTG-Ab assay better performances in sensibility, specificity and correlation with the degree of intestinal lesion.

## Conclusions

In conclusion, histology remains the corner stone for diagnosis of CD and our data demonstrate that the IgA-AAA assay is not useful in the standard work-up for the diagnosis of CD, particularly because it is not able to identify mild and subtotal villous atrophy. Nevertheless, the IgA-AAA presence suggests that the ingestion of gluten had already caused advanced intestinal mucosal lesions and that IgA-AAA measurement could have a role to support the diagnosis of CD when the histology interpretation is difficult (i.e. patchy distribution), when the biopsy is avoided for clinical reasons (i.e. when a biopsy or anesthesia represents a life-threatening risk), or in case of negative parents' consensus to endoscopy.

## Abbreviations

CD: Celiac disease; IgA-AAA: Antibodies Anti-Actin filaments IgA; tTG-Ab: anti-tissue transglutaminase antibodies; M/O: Marsh/Oberhuber; IFI: indirect immunofluorescence; ELISA: Enzyme-Linked Immunoabsorbent Assay; EmA: anti-endomysium.

## Competing interests

The authors declare that they have no competing interests.

## Authors' contributions

EB conceived of the study, carried out the immunoassays, participated in the coordination of the study and polished the manuscript. BP conceived of the study, selected patients, coordinated the study and drafted the manuscript. GMT and PC participated in the design of the study and drafted the manuscript. LA evaluated histological intestinal specimens. SM performed the statistical analysis. GB participated in the coordination of the study.

All authors read and approved the final manuscript.
